# Histamine 50-Skin-Prick Test: A Tool to Diagnose Histamine Intolerance

**DOI:** 10.5402/2011/353045

**Published:** 2011-02-22

**Authors:** Lukas Kofler, Hanno Ulmer, Heinz Kofler

**Affiliations:** ^1^Private Allergy Clinic Hall i.T., Thurnfeldgasse 3a, 6060 Hall i.T., Austria; ^2^Department of Medical Statistics, Informatics and Health Economics, Medical University of Innsbruck (MUI), Schöpfstraße 41/1, 6020 Innsbruck, Austria

## Abstract

*Background*. Histamine intolerance results from an imbalance between histamine intake and degradation. In healthy persons, dietary histamine can be sufficiently metabolized by amine oxidases, whereas persons with low amine oxidase activity are at risk of histamine toxicity. Diamine oxidase (DAO) is the key enzyme in degradation. Histamine elicits a wide range of effects. Histamine intolerance displays symptoms, such as rhinitis, headache, gastrointestinal symptoms, palpitations, urticaria and pruritus. *Objective*. Diagnosis of histamine intolerance until now is based on case history; neither a validated questionnaire nor a routine test is available. It was the aim of this trial to evaluate the usefullness of a prick-test for the diagnosis of histamine intolerance. *Methods*. Prick-testing with 1% histamine solution and wheal size-measurement to assess the relation between the wheal in prick-test, read after 20 to 50 minutes, as sign of slowed histamine degradation as well as history and symptoms of histamine intolerance. *Results*. Besides a pretest with 17 patients with HIT we investigated 156 persons (81 with HIT, 75 controls): 64 out of 81 with histamine intolerance(HIT), but only 14 out of 75 persons from the control-group presented with a histamine wheal ≥3 mm after 50 minutes (*P* < .0001). *Conclusion and Clinical Relevance*. Histamine-50 skin-prickt-test offers a simple tool with relevance.

## 1. Introduction

In a series of experiments [1], two groups of anesthetized pigs were administered cheeses via gastric tube. While one group showed no symptoms after anesthesia, pigs of the other group succumbed in severe anaphylactic shock. The only prior intervention in this group consisted in irreversibly blocking the histamine degrading key enzyme diaminooxidase (DAO) before feeding cheese, rich in histamine. As a result, the histamine in cheese could not be metabolized and reached a level comparable to fatal allergic anaphylactic shock. These key experiments for the first time displayed the importance of hindered histamine metabolism and proved the concept of “luminal-induced enteral histaminosis” [1]. 

Histamine induced symptoms in a growing number of patients, with strong belief in, albeit no evidence for, underlying allergy has generated growing interest. For the classification of these symptoms, the term “histamine intolerance (HIT)” has become popular [2, 3]. It is assumed that histamine intolerance results from an imbalance of exogenous histamine intake and/or histamine metabolism due to impaired enzymatic effect for a variety of reasons. The clinical picture of gastrointestinal signs of HIT is sometimes puzzled by concomitant lactose intolerance and/or fructose malabsorption. It is presumed that histamine intolerance concerns 1–4% of the general population [2].

Histamine (MW 111) is a small biomolecule, but one of the most extensively studied entities, having at least 23 different physiological functions [4]. Histamine, 2-(4-Imidazolyl-)ethylamine, is the biogenic amine from histidine that serves as substrate for its rate-limiting enzyme histidine decarboxylase (HDC). Histamine is mainly produced in mast cells and basophils, stored in many tissues and released upon a variety of immunological [5] and nonimmunological signals [2, 5]. In the body, formation of histamine takes place in one step. HDCs widely contain pyridoxal phosphate (PLP) as cofactor. PLP-dependent HDCs are ubiquitous; they can be found not only in mammalian tissue but also in numerous bacteria. Elevated concentration of histamine in some processed foods is explained through contamination from several bacteria and their HDC activity. The metabolic pathways are different in central nervous system (CNS) and the periphery, with an extra 3-methylation step in the brain. Both types of transformation result in derivatives of imidazole -acetic acid with enhanced water solubility and are excreted in the urine. Histamine is a key mediator of allergic and nonallergic diseases and plays its role in allergic rhinitis, urticaria, and anaphylaxis and in bronchial asthma [1, 5]. As mentioned, DAO converts amines leading to the formation of the final products. At least for putrescine, there is evidence that DAO is responsible for conversion also in vivo. In brief, an assay for DAO activity is based on the conversion of [1, 4-^14^ C]putrescine to 4-amino-[1, 4-^14^ C] butylaldehyde which in alkaline solution spontaneously forms Δ1-[1, 4-^14^ C]pyrroline that can be extracted into an organic solvent for quantization [6]. This assay has been widely used for diagnosis of histamine intolerance and diminished DAO levels in plasma imputed to histamine intolerance [6, 7]. However, this remained un successful as these results could not be confirmed by others [8, 9]. So, only careful history taking of a patient's symptoms until now confirms the clinical diagnosis of histamine intolerance. A simple diagnostic test was investigated and named “Histamine 50-prick-test.”

## 2. Material and Methods

**Figure 1:**
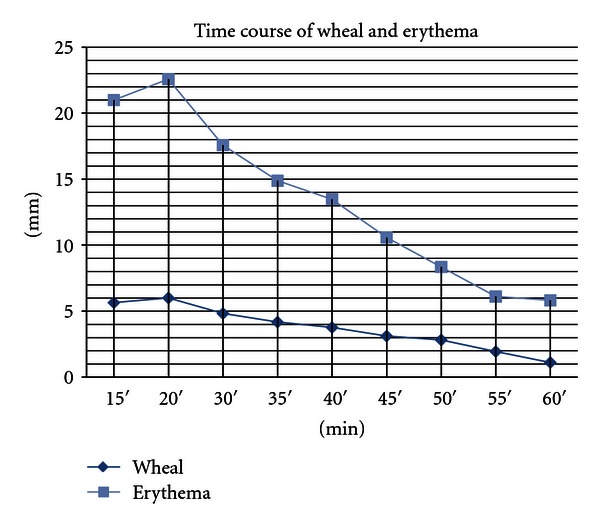
*n* = 17 patients with histamine intolerance: skin prick test wheal size read from 15 to 60 minutes. Test substance: “pangramin prick positive control: active agent: 1% histamine dihydrochloride,” solution, (10 mg/ml), cut off level ≥ 3 mm.

Skin prick testing is an established technique for diagnosis of type-I allergic reactions such as inhalant allergens, food, or drugs on intact skin [10, 11]. For standardization a positive and a negative control is included in each test [11]. 0.9% of physiologic saline solution serves as a negative control, whereas aqueous 1% (10 mg/ml) of histamine hydrochloride solution is used as positive control throughout all tests. It is carried out on the volar surface of the lower arm. Likewise a drop of saline solution and histamine solution (Pangramin Positivkontrolle ALK-Abello Österreich, “Histamindihydrochlorid, Lösung,” 1% (10 mg/ml, also contains phenol, glycerol, potassium phosphate, potassium chloride, aqua ad inj.) were pipetted on the intact skin and pricked with lancets (M Mediware, Blutlanzette, steril, Premium Quality, REF B2 01). Asymmetrical wheals were measured as follows: wheal size perpendiculars to each other were measured, divided by 2 and the average wheal diameter used in millimeter (mm). Testing was always performed by two investigators (technicians M. E. and M. S.) and results at various time intervals recorded by two investigators (LK and HK) blinded to which group a test person belonged. Usually, skin prick tests are read after 20 minutes. In a pretest, 17 extra test persons with known histamine intolerance were closely monitored; the diameter of wheal and erythema was measured every 5 minutes until 60 minutes after skin prick (Figure 1). From this, we considered a wheal ≥3 mm up to 50 minutes as positive. 

 This study comprised 156 probands, (male = 62, female = 94); mean age was 29 (control group) and 39 (histamine intolerant group), respectively. All gave their written consent to participate in this study. All of them were outpatients of the private allergy clinic for different reasons and underwent routine skin prick tests. Patients with known inflammatory bowel diseases (Crohn's disease, colitis ulcerosa), irritable bowel syndrome (IBS), and celiac disease had been excluded to participate, as were patients with prior corticosteroid or H1 blocker pretreatment within the last 4 weeks.

In all 156 patients a thorough case history was taken. On the basis of their, often longstanding symptoms (i.e., headache, palpitations, diarrhea, and pruritus after alcoholic beverages or food, known to be rich in histamine) and physical investigation, we categorized two groups: 81 patients with presumable histamine intolerance and a control group consisting of 75 patients attending the allergy clinic from urticaria, pollen-, or mite-allergy but no history nor any anamnestic signs of histamine intolerance.

## 3. Statistics

For analysis of frequency distribution, contingency tables and Pearson's Chi-square test were used. Data of sensitivity, specificity, positive and negative predictive value and their 95% confidence intervals were calculated (Table 1). Best discrimination time point between histamine group and control group was calculated from an ROC analysis. Levels of significance were set at  0.05.

## 4. Results

**Figure 2:**
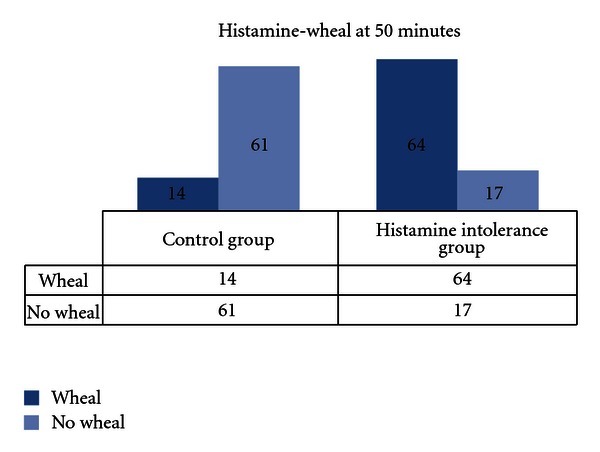
HIT group *n* = 81, control group *n* = 75; wheal size was read 50 minutes after testing with a histamine standard prick-test, 78 /156 probands displayed a histamine-wheal ≥ 3 mm, 17,95% (*n* = 14) from the control group and 82,05% (*n* = 64) from the HIT group.

**Figure 3:**
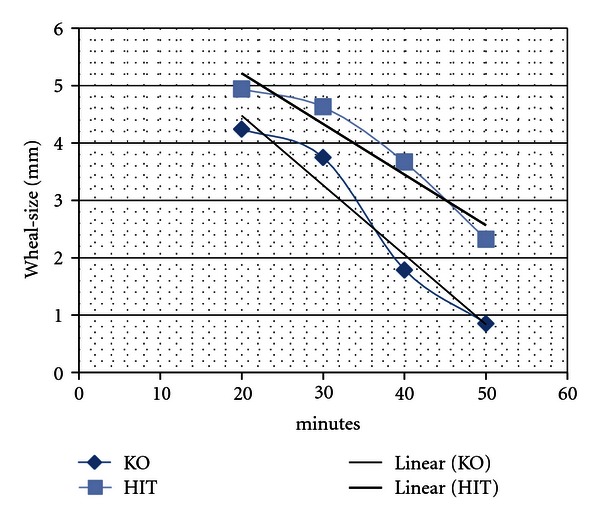
Control group = 75, HIT group = 81. Wheal size and time course of a histamine-wheal in the control-group and the HIT group; measured at 20, 30, 40, and 50 minutes.

(1) ROC analysis showed that best discrimination between histamine and control group is displayed at 50 minutes. Here 50% (*n* = 78) of all test persons still had a wheal ≥ 3 mm; 17.95% (*n* = 14) of them were from the control group, but 82.05% (*n* = 64) from the histamine intolerance group (Figure 2). Sensitivity of the histamine 50-skin-prick test is 79% (95% confidence intervals: 68.5–87.3%) and specificity is 81.3% (95% confidence intervals 70.7%–89.4%). The negative predictive value of this test is 78.2% (67.4–86.8%) and the positive predictive value is 82.1% (71.7.5–89.8%).

(2) No differences could be observed between either sexes of patients nor between different ages.

(3) Wheal diameter peaked at 20 minutes and was slowly diminishing to minute 60 (Figure 3).

## 5. Discussion

In most publications [8–10, 12–14] on histamine intolerance until so far, unvalidated questionnaires for diagnosis have been used, simply as no validated questionnaires are published or available. Histamine is a pleiotropic [4] substance, the imbalance of histamine intake and metabolism probably leads to the wide spectrum of symptoms in histamine intolerance. Key enzyme in histamine metabolism seems to be DAO. Its expression is high in intestine, kidney and placenta. The substrate specificity of amine oxidases is limited, low DAO activity might be due to the presence of large amounts of other amineoxidases [3]. Therefore data on the expression of DAO based solely on activity measurements should be considered cautiously. It was clearly shown in patients with histamine intolerance that plasma DAO activity could not be correlated to histamine intolerance [8, 9]. Histamine is metabolized in the skin [15]. An experiment, measuring histamine degrading enzyme acticivity of (healthy) human skin homogenates, displayed that the main histamine degrading enzyme was N-methyl-transferase, not DAO. This study, however, was not aimed to evaluate the histamine degrading capacity of skin in HIT patients. It is widely assumed, that a double blind oral provocation test with varying amounts of an aqueous histamine solution would serve as “gold standard” for diagnosis. Besides its expense in daily practice, oral provocation with histamine is very difficult to standardize. It has been published that even in patients with overt histamine intolerance oral provocation was positive in only 50% of those tested [14]. Very recently a multicenter study on the nonreliability of blinded oral histamine provocation to confirm histamine intolerance has been published [16]. Obviously oral histamine provocation test thus cannot be considered a gold standard in HIT. To date, diagnosis; therefore, is entirely based on history taking and post hoc affirmation by successful dietary measures. A link between histamine and altered mental state has been published. In Kounis-syndrome, among others, depression is said to activate not only a cascade of inflammatory mediators like TNF-alfa, but also triggers mast cells to release histamine: ultimately this leads to symptoms of a coronary syndrome without morphological abnormalities in affected patients [17, 18]. Ginsburg studied 12 patients with nonexertional chest pain who were given intravenous histamine. They demonstrated histamine to be capable of inducing coronary artery spasm [18].

The mast cells are the major effector cell in immediate hypersensitivity Also the growing importance role of histamine in the amine system in the brain and augmented peripheral histamine effects caused by stress has been elucidated [19].

A lot is known about histamine; less is known about histamine intolerance in the medical community, and the scientific evidence to date is scarce. Only recently the following has been stated: “*Evaluation of more than 200 scientific journal articles and over 30 patient oriented websites dealing with this disease concept Diamine oxidase (DAO) revealed that a lot more is being alleged and stated than is actually substantiated by scientific evidence*” *[3]*. The concept of HIT implies that altered histamine metabolism must not only occur in the gastrointestinal tract but in all tissues where signs of HIT can be observed. Regarding histamine metabolism in the skin, remarkably enough, only few data, for example, from animal studies [20], are published so far. These authors could show an impaired histamine metabolism in SLE mice.

Francis et al. [15] were among the first to study the role of histamine metabolism in skin in 1977. Certainly this awaits further elucidation in future studies, which was beyond the scope of this clinical study, however.

Almost all of these clinically relevant effects are mediated through H1 receptors, whereas H2 receptors can be mainly found in gastric, cardiac and some gland tissue. Therefore, most acute histamine effects can adequately be treated with H1 antihistamines. It is known that mastocytosis [21] as well as exogenous histamine from food—according to its amount—can lead to identical symptoms. Processed foods, often rich in histamine (such as Parmesan cheese, canned fish, and rehashed food), histamine liberating foods,although discussed with controversy [10, 12, 13], alcohol [22, 23] that blocks DAO and may contain histamine such as some red wines and certain drugs, releasing histamine [24–27] from mast cells such as radiocontrast agents or NSAIDs, point to the role of histamine as mediator that mimics some features of allergic disease. However, more compelling evidence for the role of histamine comes from observations that specific receptor blockade and dietary restriction leads to long-lasting improvement of symptoms in most of these patients. Another intriguing observation can be made during pregnancy of female patients; during the third trimester the placenta produces many times more DAO than it is detectable in nonpregnant women. The improvement of histamine intolerance in many women, although only during their late pregnancy, may support this hypothesis [28]. Genetic background might play a certain role; various single nuclear polymorphisms (SNPs) for DAO have been described [29, 30] on chromosome 7. The importance of epigenetics on histamine intolerance is on the dice but has not systematically been investigated until now.

The diagnostic uncertainty led us to investigate the usefulness of the histamine skin prick test with readings at 50 minutes, however. We showed, that patients with histamine intolerance and a control group do not remarkably differ in the size of their histamine wheals (Figure 3), but remarkably in their time course of the histamine wheals ≥3 mm read at different time points (*P* < .0001). This difference in skin prick test allows discriminating for histamine intolerance with sufficient sensitivity and specificity. This test is simple and can be used without any adaption in each office where skin prick testing is performed.

## 6. Conclusion

Combined with a thorough history taking, a sound diagnosis of histamine intolerance can easily be achieved with a histamine 50-skin-prick test.

##  Funding/Financial Disclosures

This study was not supported by any funding. None of the authors received neither any financial support nor any consultation fees.

## Figures and Tables

**Table 1:** Descriptive statistics.

Statistics	Figure	Lower 95%-confidence intervall	Upper 95%-confidence intervall
Positive- predictive value	0,82051	0,71723	0,89825
Negative- predictive value	0,78205	0,67414	0,86761
Sensitivity	0,79012	0,68537	0,87272
Specificity	0,81333	0,70669	0,89402
